# Boar seminal plasma exosomes maintain sperm function by infiltrating into the sperm membrane

**DOI:** 10.18632/oncotarget.11315

**Published:** 2016-08-16

**Authors:** Jian Du, Jian Shen, Yuanxian Wang, Chuanying Pan, Weijun Pang, Hua Diao, Wuzi Dong

**Affiliations:** ^1^ College of Animal Science and Technology, Northwest A&F University, Yangling, Shaanxi, P. R. China; ^2^ NPFPC Key Laboratory of Contraceptives and Devices, Shanghai Institute of Planned Parenthood Research (SIPPR), Institutes of Reproduction and Development, Fudan University, Shanghai, P. R. China

**Keywords:** seminal plasma exosomes, boar sperm quality, capacitation, liquid storage, Pathology Section

## Abstract

Seminal plasma ingredients are important for maintenance of sperm viability. This study focuses on the effect of boar seminal plasma exosomes on sperm function during long-term liquid storage. Boar seminal plasma exosomes had typical nano-structure morphology as measured by scanning electron microscopy (SEM) and molecular markers such as AWN, CD9 and CD63 by western blot analysis. The effect on sperm parameters of adding different ratio of boar seminal plasma exosomes to boar sperm preparations was analyzed. Compared to the diluent without exosomes, the diluent with four times or sixteen times exosomes compared to original semen had higher sperm motility, prolonged effective survival time, improved sperm plasma membrane integrity (*p* < 0.05), increased total antioxidant capacity (T-AOC) activity and decreased malondialdehyde (MDA) content. The diluent containing four times concentration of exosomes compared to original semen was determined to inhibit premature capacitation, but not to influence capacitation induced *in vitro*. Inhibition of premature capacitation is likely related to the concentration of exosomes which had been demonstrated to transfer proteins including AWN and PSP-1 into sperm. In addition, using fluorescence microscopy and scanning electron microscopy analysis, it was demonstrated that exosomes in diluent were directly binding to the membrane of sperm head which could improve sperm plasma membrane integrity.

## INTRODUCTION

In order to become fully fertile after leaving testis, mammalian sperm must undergo morphological and functional changes during transit through epididymis and other accessory sex organs. Specifically, proteins and lipids on the sperm membrane are subtly modified by secretions of the male genital tract [1]. Upon ejaculation, spermatozoa are mixed with another set of surface re-modeling components derived from the accessory sex glands. Secretions from the male genital tract can influence the capacitation of sperm during their transit along the female genital tract [2]. Molecules on the surface of spermatozoa and in seminal plasma act in concert to stimulate or inhibit the onset and progression of the capacitation process [3, 4]. It is thought that early capacitation-related events are accompanied with the loss, modification and redistribution of molecules on the sperm surface during sperm storage *in vitro* [5, 6]. The decapacitation factors on sperm membranes and in seminal plasma are believed to stabilize the sperm membrane and keep sperm in a non-capacitated state, until these factors are removed during capacitation. It has also been observed that addition of ram or boar seminal plasma to sperm preparations improved viability and motility and reduced capacitation-like changes during sperm storage. Therefore, it is necessary to identify the components of boar seminal plasma that help to maintain sperm function during sperm storage *in vitro*.

Seminal plasma vesicles, which are characterized by high enrichment of cholesterol and sphingomyelin content, and a complex protein composition, have been isolated from the seminal plasma of human (namely prostasomes) [2, 7], rat [8], ram [9], and boar [10]. It has been hypothesized that mammallian seminal plasma vesicles (perhaps including exosomes) can mediate decapacitation activity using several possible mechanism, such as inhibiting the capacitation-dependent cholesterol efflux, fluidity increase in sperm apical membranes, delivery of Ca^2+^ signaling molecule, and control of the acrosome reaction [11-13].

Seminal plasma exosomes are membrane vesicles ranging from 30 to 120-nm diameter in size that are produced by organs in the male genital tracts including epididymis and prostate [10, 14, 15]. In boar, seminal plasma exosomes could inhibit the capacitation-dependent cholesterol efflux and mediate degradation of the 14-kD phosphorylated polypeptide of capacitated sperm [10, 12]. Several previous studies have demonstrated that seminal plasma exosomes may play an important role in maintaining the sperm function during *in vitro* preservation. However, the effect of boar seminal plasma exosomes as an additive to maintain semen quality during preservation at low temperature has not been reported. In the present study, the exosomes were isolated from Guanzhong-Black boar. Then the effect of exosomes on sperm motility, sperm membrane integrity, lipid peroxidation, and capacitation were investigated. Finally a possible modulatory role of exosomes on sperm function was firstly determined during long-term liquid storage of boar sperm at 17^o^C was discussed.

## RESULTS

### Enrichment of boar seminal plasma exosomes

To concentrate and identify boar seminal plasma exosomes, boar seminal plasma exosomes were isolated by ultracentrifugation using modified methods as previously described [10, 14]. The exosome concentration of original seminal plasma (in Part I) were about 2×10^10^ particles/mL as calculated by the NanoSight system. After concentration of these samples was calculated, different final exosome concentration in the diluent were adjusted to the exosomes concentration of original seminal plasma in the following experiments.

**Figure 1:**
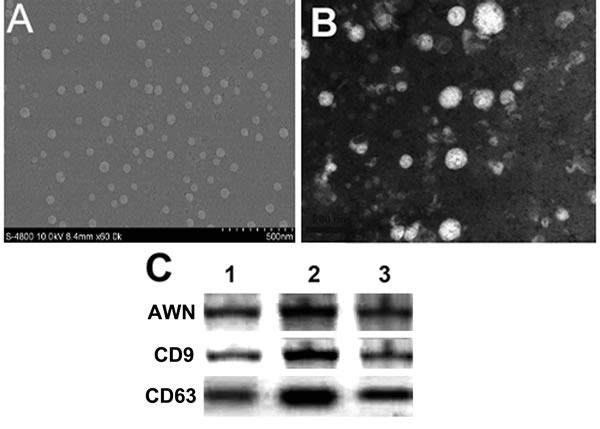
Identification of boar seminal plasma exosomes **A.**, **B.** Morphology of exosomes by scanning electron microscope. Morphology of negative stained exosomes (B); **C.** Molecular markers of exosomes (AWN, CD9 and CD63) were detected by Western blot. 1, 2, 3 lanes represented three repetitions.

SEM demonstrated that some spherical membranous structures in the sample were 50-100 nm diameter membranous vesicles, within the size range of typical exosomes (Figure 1A, 1B). Furthermore, AWN, CD9 and CD63 in the membranous vesicles were proved by Western blot (Figure 1C).

### Boar seminal plasma exosomes prolonged effective motility time of sperm

After the particle concentration was adjusted to the exosomes concentration of original seminal plasma, the effect of different concentrations of exosomes on boar sperm motility during liquid storage at 17°C was measured (Table 1). Sperm motility remained above 50% in the diluent (Exo-0) after 6 day of storage. For samples supplemented with Exo-1, Exo-4 and Exo-16 exosome additives, sperm motility was significantly higher than that of other groups after 8 days of storage (*p* < 0.05). The samples supplemented with Exo-4 exosomes maintained the highest motility of all groups after 10 days of storage (*p* < 0.05), but there was no difference between the groups with Exo-4 and Exo-16 addition (*p* > 0.05). These results indicated that Exo-4 and Exo-16 exosomes supplementation resulted in significantly prolonged sperm motility during storage.

**Table 1:** Effects of different concentrations of semen plasma exosomes on boar sperm motility (%)

Conservation Time/day	Different concentration of semen plasma exosomes			
	Exo-0	Exo-1	Exo-4	Exo-16
0	83.69±2.46	84.23±2.42	84.012±3.95	85.50±3.52
2	69.33±1.23 b	70.17±1.89 ^a^^b^	72.00±0.50 ^a^	69.33±2.82^b^
4	58.33±1.25 d	65.67±1.74 ^c^	71.17±1.45 ^a^	69.67±2.54 ^b^
6	50.30±1.89 c	60.00±1.21 ^b^	64.12±1.53 ^a^	64.17±1.76 ^a^
8	41.84±1.53 d	56.33±1.23 ^c^	62.00±0.96 ^a^	60.17±0.87 ^b^
10	30.25±2.35 c	49.50±1.50^b^	56.27±1.75 ^a^	49.00±1.43 ^b^

Different alphabets superscripts within the same row demonstrate significant differences (*p*<0.05), and the same alphabets superscripts demonstrate not significant (*p*>0.05). Exo-0, Exo-1, Exo-4, Exo-16 represented the diluent with 0, 1 4 and 16 times concentration of exosomes of original semen, respectively.

### Boar seminal plasma exosomes improved plasma membrane integrity of spermatozoa

The effects of different concentrations of exosomes on boar sperm plasma membrane integrity during liquid storage at 17°C is shown in Table 2. After 2 days of storage, the plasma membrane integrity of the samples supplemented with Exo-1, Exo-4, and Exo-16 exosomes were significantly higher than that of other samples (*p* < 0.05). Furthermore, after 10-day of storage, samples supplemented with Exo-4, and Exo-16 exosomes maintained more than 60% plasma membrane integrity, which was significantly higher than that of other groups (*p* < 0.05). However, there were no significant differences between the Exo-4 and Exo-16 samples (*p* > 0.05). The results indicated that supplementation with high concentration of exosomes (to at least the concentration of Exo-4) significantly improved the plasma membrane integrity of spermatozoa during long-term liquid storage at 17 °C.

**Table 2:** Effects of different concentrations of semen plasma exosomes on boar sperm plasma membrane integrity (%)

Conservation Time/day	Different concentration of semen plasma exosomes			
	Exo-0	Exo-1	Exo-4	Exo-16
0	90.50±2.46	91.11±4.38	92.43±2.95	92.16±2.48
2	79.36±2.94 ^c^	83.83±1.81 ^d^	85.22±2.62 ^a,b^	87.43±3.16^a^
4	71.21±2.53 ^c^	77.92±1.53 ^d^	80.47±3.30 ^a^	81.07±2.50 ^a^
6	61.84±3.35 ^c^	69.89±3.95 ^d^	75.15±3.46 ^a^	74.37±1.63 ^a^
8	41.96±1.18 ^c^	61.27±2.40 ^d^	70.95±1.92 ^a^	72.58±3.23 ^a^
10	35.20±2.88 ^c^	55.30±3.39 ^d^	66.10±3.94 ^a^	67.96±3.74 ^a^

Different superscripts within the same row demonstrate significant differences (*p*<0.05), and the same superscripts demonstrate not significant (*p*>0.05). Exo-0, Exo-1, Exo-4, Exo-16 represented the extender with 0, 1, 4 and 16 times concentration of exosomes of original semen, respectively.

### Determination of T-AOC activity and MDA content of sperm

**Figure 2:**
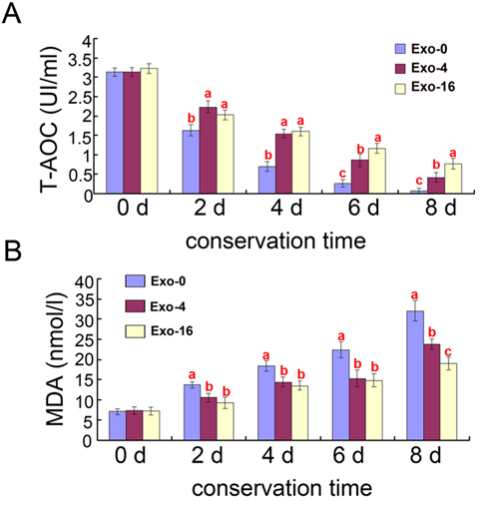
Effects of different concentrations of exosomes on T-AOC and MDA content in boar sperm during liquid storage at 17°C **A.** Effects of different concentrations of exosomes on T-AOC activity. **B.** Effects of different concentrations of exosomes on MDA content. Different superscripts within the same time demonstrate significant differences (*p* < 0.05), and the same superscripts demonstrate insignificant differences (*p* > 0.05). Exo-0, Exo-4, Exo-16 represented the diluent with 0, 4 and 16 times concentration of exosomes of original semen, respectively.

In order to analyze the antioxidant capacity of exosomes, T-AOC activity and MDA content of the samples supplemented with Exo-0, Exo-4, Exo-16 exosomes were determined during long-term liquid storage at 17°C, respectively (Figure 2). Compared to samples without exosomes supplementation, decreased T-AOC activity and increased MDA content was observed in the groups supplemented with Exo-4 and Exo-16 (*p* < 0.05). T-AOC activity of the groups supplemented with Exo-4 was significantly lower after 6 days of incubation (*p* < 0.05) (Figure 2A) and MDA content was significantly higher after 8 day of incubation (*p* < 0.05) (Figure 2B) compared to samples with Exo-16 exosomes supplementation, respectively. The decrease of T-AOC activity and increase of MDA content accompanied by an increase of seminal plasma exosomes content indicated that Exo-4 might be the optimal concentration of exosomes for storage of sperm samples at 17°C.

### Effect of boar seminal plasma exosomes on *in vitro* sperm premature capacitation

**Figure 3:**
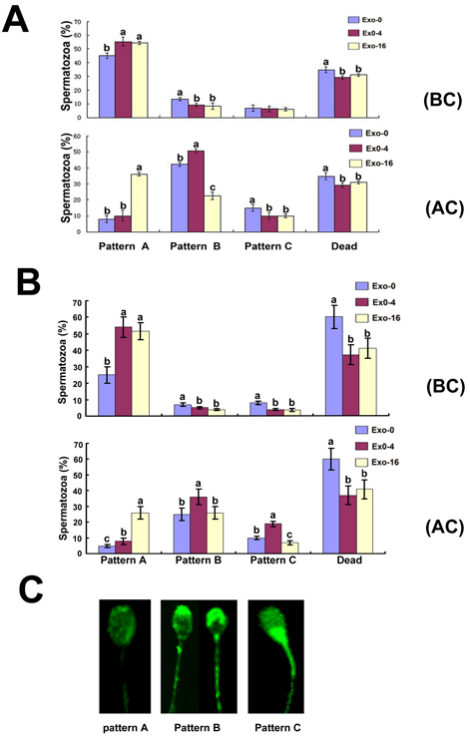
CTC staining patterns of sperm in the diluent with Exo-0, Exo-4 and Exo-16 exosomes A, CTC staining patterns of sperm on the 4^th^ day in the diluent with Exo-0, Exo-4 and Exo-16 exosomes. (BC): before capacitation; (AC): at 3 hours after sperm capacitation was induced. B, CTC staining patterns of sperm on the 8^th^ day in the diluent with Exo-0, Exo-4 and Exo-16 exosomes. Capacitation fluid was Tyrode's medium with 3 mg/mL BSA (see Materials and Methods). (BC): before capacitation; (AC): at 3 hours after sperm capacitation was induced. C, illustration of spermatozoa different fluorescence patterns of spermatozoa samples. Pattern **A.** faint fluorescence uniformly distributed over the sperm head denoting non-capacitated spermatozoa. Pattern **B.** the acrosomal region of the sperm heads fluoresce brightly denoting capacitated spermatozoa. Pattern **C.** the acrosomal region of the sperm head is non-fluorescent while the post-acrosomal region of the sperm head appeared strong fluorescence and this indicated a capacitated, acrosome-reacted spermatozoa. Dead represented the percentage of spermatozoa with no movement of the flagellum. Exo-0, Exo-4, Exo-16 represented the diluent with 0, 4 and 16 times concentration of exosomes of original semen, respectively. Sperm head length: 7.0 μm.

In order to analyze the effects of exosomes on sperm capacitation *in vitro*, CTC staining was used to determine fluorescent patterns of sperm in the groups supplemented with Exo-0, Exo-4, and Exo-16 exosomes on day 4 of storage before and after sperm capacitation was induced using Tyrode's medium containing 3 mg/mL bovine serum albumin (BSA) at 37°C (Figure 3A). Before capacitation, the percentage of CTC staining fluorescent pattern A of the samples without exosomes was lower and that of pattern B was higher than other groups, but no difference was observed for pattern C. After capacitation, pattern A of sperm in the groups with Exo-16 exosomes was the highest and pattern B was the lowest compared with other groups, (*p* < 0.05). Notably, the rate of pattern A to pattern B,C was not different between the groups with Exo-4 exosomes and the groups without exosomes, although pattern B of sperm in the groups with Exo-4 exosomes was the highest than others after induced capacitation. The same results appeared on day 8 of sperm storage *in vitro* (Figure 3B). These results indicated that exosomes could inhibit premature capacitation of sperm.

### Analysis of AWN and PSP-1 on sperm during *in vitro* storage

**Figure 4:**
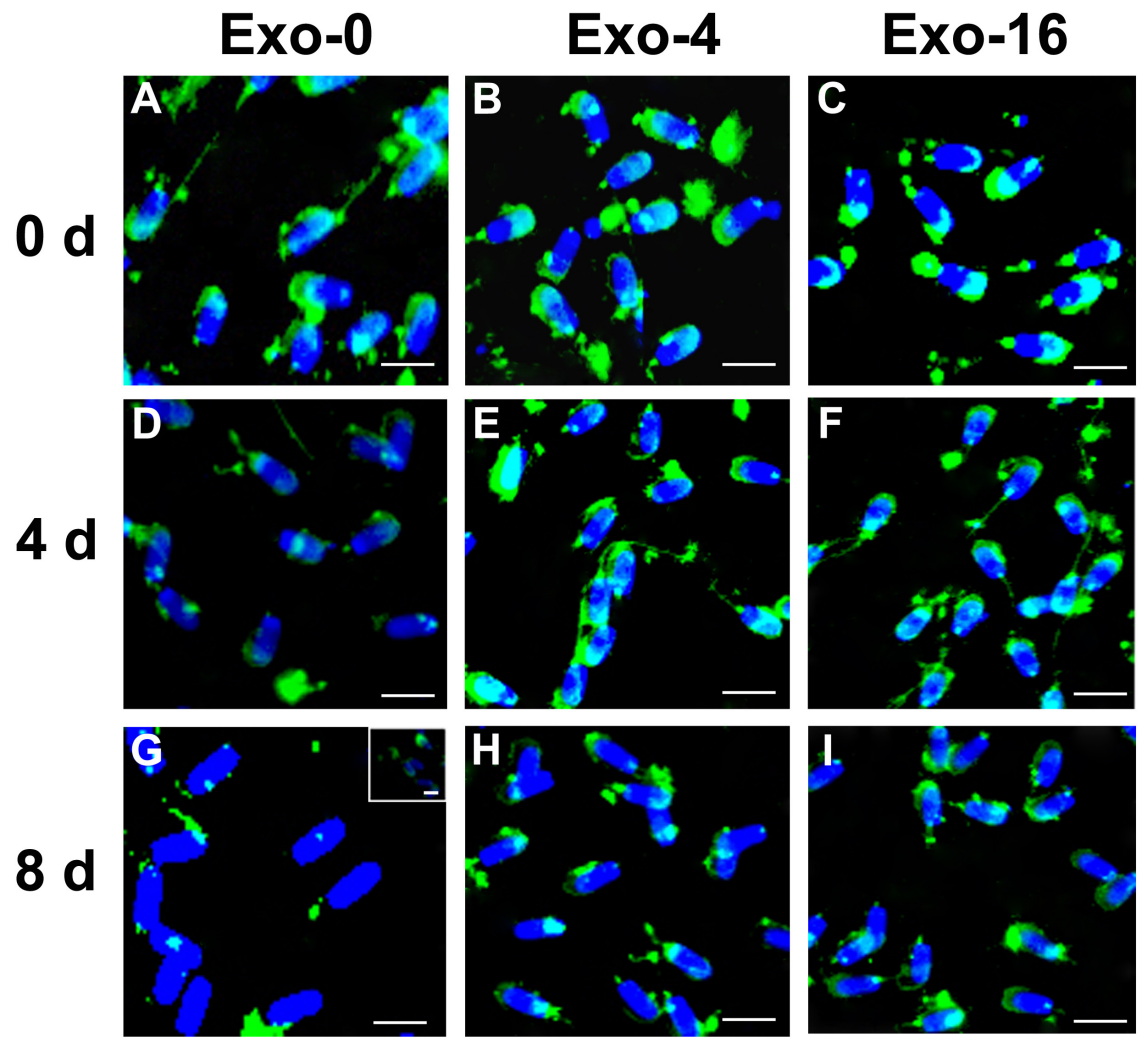
Immunofluorescence detection of AWN on sperm incubated in the diluent without exosomes (Exo-0) and with different concentrations of exosomes (Exo-4 and Exo-16) on day 0, and after 4 days and 8 days at 17°C In the diluent without exosomes (Exo-0), AWN was present on the acrosome membrane of all sperm on day 0 **A.** Partial AWN protein existed on acrosome membrane of sperm **D.** All sperm had lost AWN on acrosome membrane by day 8 **G.** In the diluent with different concentrations of exosomes (Exo-4 and Exo-16), respectively, AWN located on the acrosome membrane of all sperm on day 0 **B.** and **C.** and day 4 **E.** and **F.** AWN dispersed in front area of sperm heads on day 8 **H.** and **I.** *Arrowheads* represented AWN location on acrosome membrane. Exo-0, Exo-4 and Exo-16 represented the diluent without exosomes, the diluent with four times exosomes concentration, and sixteen times exosomes concentration of original semen, respectively. Sperm head length: 7.0 μm.

**Figure 5:**
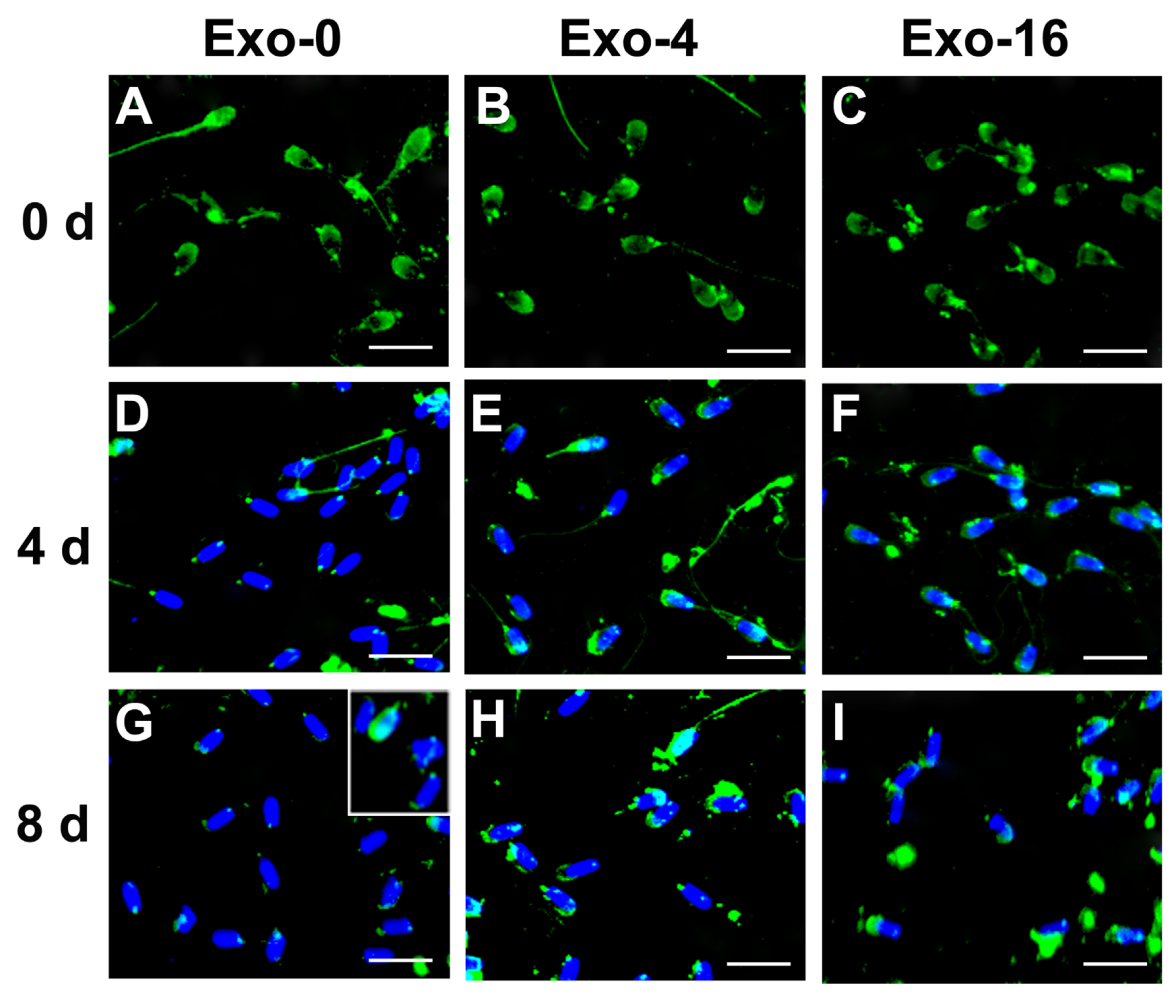
Immunofluorescence detection of PSP-I on sperm incubated in the diluent without exosomes (Exo-0) and with different concentrations of exosomes (Exo-4 and Exo-16) on day 0, and after 4 days and 8 days at 17°C In the diluent without exosomes, PSP-1 located on acrosome membrane and apical area of sperm tail on day 0 **A.** Partial PSP-1 protein existed on the acrosome membrane and principal area of sperm tail on day 4 **D.** All sperm had lost PSP-1 on acrosome membrane and only remain PSP-1 remained in the apical area of the middle piece of sperm tail on day 8 **G.**. In the diluent with different concentrations of exosomes (Exo-4 or Exo-16), respectively, PSP-1 existed on the acrosome membrane and principal areas of the sperm tail on day 0 **B.**, **C.** and day 4 **E.**, **F.** and sperm lost part of the PSP-1 on the acrosome membrane on day 8 **H.**, **I.** *Arrowheads* and *asterisk* represent PSP-1 locations on acrosome membranes and on the apical area of the middle piece of sperm tails, respectively. Exo-0, Exo-4 and Exo-16 represented the diluent without exosomes, four times exosomes concentration and sixteen times exosomes concentration of original semen, respectively. Sperm head length: 7.0 μm.

AWN and PSP-1 which are highly abundant proteins in boar seminal plasma have been observed to load in exosomes [10, 16, 17]. To analyze changes of proteins on the sperm membrane, immunofluorescence was used to detect the location of AWN and PSP-1 on sperm on day 0, day 4, and day 8 of sperm storage in the samples with Exo-0, Exo-4, or Exo-16 supplementation (Figure 4 & 5). AWN was primarily localized on the acrosome membrane (Figure 4A, 4B, 4C) as the same as previous reports [16, 17] have reported and PSP-1 proteins appeared simultaneously on acrosome membranes and in the apical area of the middle piece of the sperm tail (Figure 5A, 5B, 5C), confirming results previously reported by Caballero Ignacio et al [18]. Loss of AWN and PSP-1 from the acrosome membrane was slower in the sample with Exo-4 exosomes than in the basic diluent without exosomes (Figure 4 and Figure 5). Notably, by day 8, the majority of spermatozoa lose AWN (Figure 4G) and PSP-1 (Figure 5G) from acrosome membrane in the diluent, but partial recovery of AWN and PSP-1 on the sperm membrane was observed when exosomes was added into the diluent for 2 days (Figure 4G insert and Figure 5G insert). Conversely, PSP-1 protein remained only on the apical area of the middle piece of sperm tail (Figure 5). However, remnants of AWN and PSP-1 still appeared on acrosome membranes on day 8 when sperm was incubated in the basic diluent with Exo-4 or Exo-16 (Figure 4 and Figure 5).

### Analysis of binding of boar seminal plasma exosomes with spermatozoa

**Figure 6:**
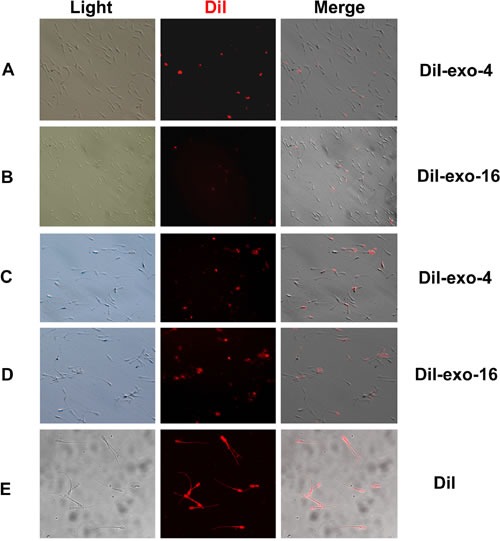
Red fluorescence sperm was detected during incubation with different concentration of DiI-exo (DiI-exo-4 and DiI-exo-16) at 17°C The sperm samples were treated by 0.125% trypsin and then washed 3 times using the diluent. Finally, the 2 μL of sperm samples were dropped on cover slides and observed using fluorescence microscopy. **A.** Sperm was incubated one day in the diluent with DiI-exo-4; **B.** Sperm was incubated one day in the diluent with DiI-exo-16; **C.** Sperm was incubated 4 days in the diluent with DiI-exo-4; **D.** Sperm was incubated 4 days in the diluent with DiI-exo-16. **E.** Sperm incubated 3 hours in the diluent with 0.005 mM DiI dye displayed red fluorescence. DiI-exo-4, DiI-exo-16 represented the diluent with four and sixteen times concentration of exosomes of original semen, respectively. And the exosomes were labeled DiI dye. Scale: sperm head length 7.0 μm.

To analyze binding or fusing of exosomes with spermatozoa, the sperm samples were observed using fluorescence microscopy after treatment by 0.125% trypsin. Spermatozoa with DiI-exo display red fluorescence under fluorescence microscope (Figure 6). Few sperm were observed with fluorescent staining in the diluent with DiI-exo (Exo-1) exosomes (data not shown). For sperm samples incubated in the diluent containing DiI-exo (Exo-4 or Exo-16) at 17°C, about 50% of the sperm showed fluorescence after one day of storage (Figure 6A, 6B). Furthermore, all sperm displayed fluorescence on day 4 of storage (Figure 6C, 6D). These results showing that binding of exosomes with spermatozoa is positively correlated to the concentration of exosomes and incubation time indicate that exosomes may directly bind to the sperm membrane.

**Figure 7:**
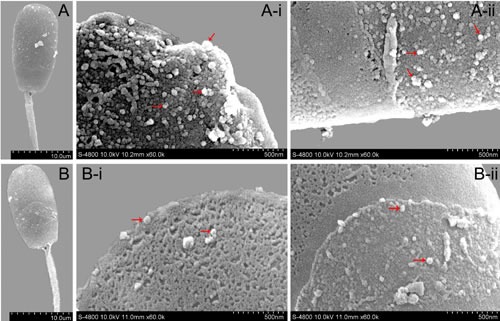
The ultrastructure of sperm at 4 d in different conditions by scanning electron microscopy (SEM) **A.** A-i, A-ii: Ultrastructure of membrane surface of sperm head in the diluent with Exo-1. **B.** B-i, B-ii: Ultrastructure of membrane surface of sperm head in the diluent with exosomes (Exo-4 or Exo-16). *Arrowheads* indicated the nano-granules (exosomes).

In order to further analyze the effect of exosomes on sperm membranes, SEM was used to observe the ultrastructure of sperm on day 4 of storage under various different conditions (Figure 7). The results of SEM showed that the number of nano-granules (exosomes) on the membrane surface of sperm in the diluent with Exo-1 were fewer than those in the diluent with Exo-4 or Exo-16 exosomes. The exosomes were found to be distributed densely on the front of sperm heads and the number of exosomes on the surface of sperm membranes was not significantly different in the diluent with Exo-4 or Exo-16.

## DISCUSSION

Mammalian seminal plasma originates from multiple cellular sources in the male genital tract including testis, epididymis, vas deferens, vesicular glands, prostate gland and other accessory sexual glands. Seminal plasma contains a high concentration of subcellular lipid-bound microparticles (extracellular vesicles) which have been identified to encompass a complex composition of lipids, mRNAs, microRNAs and proteins [12, 19]. Exosome-like vesicles have also been isolated from the seminal plasma of rat [20], rabbit [21], ram [22], bull [23], stallion [24], and boar [10]. Mammalian seminal plasma exosomes share common characteristics, though functions of the accessory sex glands vary among mammalian species and this may well affect the nature and relative proportions of the extracellular vesicles. In the current study, exosomes isolated from boar seminal plasma were lipid-bound microparticles ranging in size from 30-100 nM in diameter, similar to other reports (Figure 1A, 1B). Molecular markers such as AWN, CD63 and CD9 were also found to be present in the exosomes as demonstrated by Western blot (Figure 1C).

In all studies so far, exosomes have been shown to be cell-derived vesicles that are present in many (perhaps all) biological fluids, including urine, blood, semen, ascites, and cerebrospinal fluid [25]. It is increasingly clear that most vesicles have specialized functions and play a key role in intercellular signaling and the immune system [26]. Mammallian seminal plasma exosomes could have extensive roles in fertilization biology. Seminal plasma exosomes inhibited the capacitation-dependent cholesterol efflux and fluidity increase in apical membranes, and 14-kD polypeptide phosphorylation [10]. Human prostasomes including exosomes have been shown to play important roles in increasing sperm motility, affecting the tyrosine phosphorylation of sperm proteins and delaying acrosomal reaction, and impacting sperm-oocyte interaction by delivering related proteins to sperm [19, 27, 28]. It has been suggested that seminal plasma exosomes may play an important role in maintaining the sperm function during sperm *in vitro* preservation of sperm samples.

Liquid storage of boar semen is widely used in commercial swine artificial insemination. Sperm motility is one of the key factors in the conservation of sperm. Different additives including some single exogenous chemical reagents and endogenous components of seminal plasma added into sperm diluent could promote semen quality during cryopreservation. Interestingly, the addition of boar seminal plasma with good sperm freezability to the freezing extender can improve boar sperm cryosurvival [29]. However, the supplementation of the freezing extender with endogenous PSP-I/PSP-II did not affect post-thaw sperm survival [30]. Furthermore, human prostasomes have been shown to play an important role in increasing sperm motility [31]. In our study, boar semen plasma exosomes did improve the sperm motility during storage in 17°C (Table 1). With the optimal concentration of exosomes (Exo-4) in the diluent, the effective preservation time was longer than 10 days (motile sperm > 50%), and sperm motility remained at 65% after 8 days of storage.

The sperm plasma membrane is an outer cell structure that acts as a physiological barrier to maintain sperm architecture. Proteins and lipids in seminal plasma contribute to maintenance of sperm membrane integrity maintenance which is required for normal sperm activities and directly reflects the degree of sperm injury. Sperm membrane integrity is also highly correlated with sperm motility and survival during sperm storage [32, 33]. In our study, the plasma membrane integrity of sperm was significantly higher in diluents supplemented with exosomes than that of control samples after 2 days of storage at 17°C (*p* < 0.05), but plasma membrane integrity of the groups supplemented with Exo-4 and Exo-16 exosomes was not significantly different until 10 days' preservation (*p* > 0.05) (Table 2). These results indicate that certain concentrations of exosomes significantly improved the plasma membrane integrity of spermatozoa during long-term liquid storage.

Oxidative stress can induce peroxidation of the sperm plasma membrane to affecting the physiology of sperm [34]. The broken balance of ROS generation and scavenging activity is disrupted leading to excessive ROS generation during boar sperm storage at room temperature [35]. The decrease of sperm motility could be explained by peroxidative damage of excessive ROS. MDA, a by-product of lipid peroxidation, has been used to monitor the degree of peroxidative damage sustained by spermatozoa and T-AOC activity is another indicator to detect sperm membrane antioxidant capacity [36, 37]. The current study demonstrating increased T-AOC activity and decreased MDA content in the diluent supplemented with exosomes indicated exosomes can elevate the antioxidant capacity of sperm samples during storage (Figure 2). Exosomes concentration was also positively correlated with sperm motility (Table 1) and plasma membrane integrity of boar sperm during liquid storage (Table 2). These results indicate that exosomes could enhance antioxidation properties of sperm by maintaining the plasma membrane integrity of sperm.

Generally, an increase of Ca^2+^ influx to sperm is an indicator of characterized capacitation status [38-40]. CTC is used to detect Ca^2+^accumulation in certain organelle membranes and sperm is labeled with CTC at those parts of the surface membranes where Ca^2+^ is present above a certain concentration to allow CTC stain [41-43]. Inhibition of premature capacitation is essential to sperm survival during long-term *in vitro* sperm preservation. Compared to the sperm samples without exosomes, inhibition of sperm premature capacitation was enhanced with an increased concentration of exosomes in the diluent at 17°C (Figure 3A). Interestingly, compared to the sperm that was incubated in the diluent without exosomes for 4 days at 17°C, the capacitation of sperm with the diluent containing Exo-4 was not significantly different (*p* > 0.05),but the capacitation of sperm in the diluent with Exo-16 was significantly decreased (*p* < 0.05) when sperm capacitation was induced by 3 mg/ml BSA (Figure 3B).

Some components in seminal plasma modulate decapacitation to influence fertilization ability of spermatozoa, and are delivered via small vesicles in seminal plasma. Consecutive complement of AWN and PSP-1 to sperm could inhibit premature capacitation [44]. In our results, remnants of AWN and PSP-1 still appeared in acrosome membranes on day 8 of storage when sperm was incubated in the diluent with Exo-4, and the similar results were observed in the diluent with Exo-16 (Figure 4 and Figure 5). Changes in AWN and PSP-1 on the sperm membrane indicated that exosomes in the diluent could help to maintain sperm function perhaps via transmission of decapacitation factors (such as AWN and PSP-1) to sperm during long-term liquid storage (Figure 4 and Figure 5).

**Figure 8:**
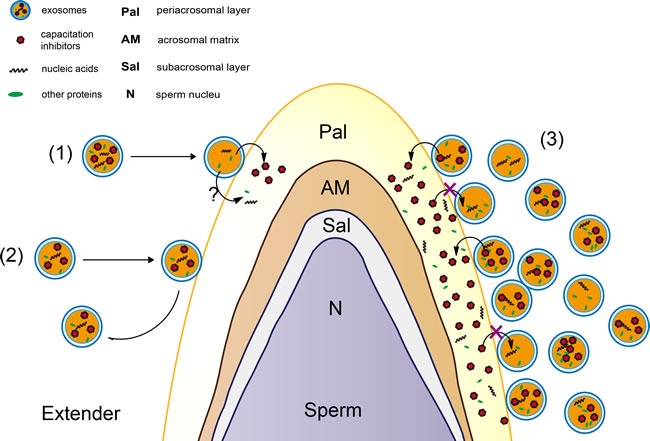
A speculative model of exosomes for protection of sperm function (**1**) It was a dynamic process that exosomes was released from and band with sperm membrane. (**2**) The elements in exosomes could be accumulated in sperm through exosomes transferring into membrane of sperm. (**3**) Excessive exosomes in diluent led to transfer more exosomes ingredients such as AWN and PSP-1 proteins (as capacitation inhibitors) into sperm membrane *in vitro*.

It is well known that cholesterol and phospholipids are essential components of mammalian cell membranes including exosomes. Consequently, cellular membrane integrity can be maintained by balancing the physiological cholesterol/phospholipid ratio [45]. Numerous studies indicate that the interaction between cholesterol and phospholipid could prevent the premature capacitation through spermatozoa membrane stabilization [46]. Vesicles (including exosomes) could inhibit progression to late capacitation events and avoid premature acrosome reaction by binding or fusion to sperm membrane early during capacitation [11]. In our study, we observed that exosomes permeate into sperm (Figure 6 and Figure 7). An interesting observation was that almost all of the sperm absorbed the DiI-exo in diluent with Exo-4 or Exo-16 exosomes after 4 days of liquid storage sperm at 17°C (Figure 6C, 6D). Furthermore, SEM revealed a large number of nano-scale granules on the sperm head membrane in the diluent with exosomes (Exo-4 or Exo-16) (Figure 7A, A-i, A-ii). In contrast, few nano-scale granules were observed on the sperm head membranes in the diluent with Exo-1 (Figure 7B, B-i, B-ii). One possible explanation is that exosomes are in a state of equilibrium inside and outside the membrane of sperm in the condition of certain concentrations of exosomes (such as Exo-4) and the balance is disturbed when excessive exosomes are added, leading to exosomes transferring more proteins into sperm including factors that inhibit capacitation (Figure 8).

## CONCLUSIONS

In this study, we report that boar seminal plasma exosomes were essential in maintaining sperm motility, sperm membrane integrity, antioxidant capacities and inhibition of premature capacitation. It was first determined that boar seminal plasma exosomes could protect sperm function through binding to the sperm membrane and transferring proteins (such as AWN and PSP-1) to sperm. Our model for exosome-mediated protection of sperm includes three speculative possibilities (Figure 8): (1) It could be a dynamic process in which exosomes are released from and bound to the sperm membrane. (2) Endogenous proteins in exosomes could accumulate in sperm through exosomal transfer. (3) Excessive exosomes in diluent could lead to transfer of more exosomal proteins such as AWN and PSP-1 into the boar sperm membrane *in vitro.* Due to the heterogeneity of exosomes derived from different organs in the genital tract, it will be necessary in future research to investigate the effect on sperm preservation of seminal plasma exosomes derived from different organs of the genital tract. It will also be interesting to determine which components in exosomes (including special proteins, lipids and miRNAs) are necessary to preserve the viability and function of porcine sperm.

## MATERIALS AND METHODS

### Semen collection

Semen samples from eight sexually mature and healthy Guanzhong-Black boars (2.5-3 years old) were used in this study. Individual ejaculates were collected into pre-warmed tubes twice a week by the gloved hand technique before transportation to the laboratory. Each sample was assessed for sperm concentration using a calibrated spectrophotometer and the percentage of motility was evaluated at 37°C using a phase-contrast microscope. Only the semen samples with more than 10^7^ spermatozoa/ml and 80% motility were selected and used for this study. The sperm-rich fractions were pooled and transferred to the laboratory within half an hour. A total of 40 ejaculates (5 ejaculate samples per boar) from eight boars were used in this study.

Semen samples were processed according to a standard cryopreservation protocol with some modifications [47]. Briefly, the semen samples were centrifuged (800 ×*g*, 10 min at room temperature) to separate sperm and supernatant. The supernatant was retained for purification of exosomes. The sperm pellets were re-suspended to a final concentration of 5×10^7^ spermatozoa/mL in the diluent supplemented with exosomes of different final concentrations at 17°C. The diluent was composed of 27.5 g of glucose, 2.35 g of EDTA-Na_2_, 6.9 g of sodium citrate, 2.9 g of citric acid, 1.0 g of NaHCO_3_, 6.65 g of Tris for per 1000 ml of deionized water (pH = 7.2) [48].

### Isolation and analysis of exosomes from boar seminal plasma

#### Isolation of exosomes from boar seminal plasma

Boar seminal plasma exosomes were isolated by ultracentrifugation according to a previously described method with modifications method of previous report [10]. In each experiment, 300 ml of the semen supernatant was centrifuged at 16,000 ×*g* for 1 h at 4°C to remove cell debris. The supernatant was filtered using 0.2 μm filters (the semen was named Part I), and centrifuged at 120,000 ×*g* for 1 hour at 4°C. The pellets were then washed twice with buffer (30 mM TRIS, 130 mM NaCl, pH 7.6) followed by centrifugation at 120,000 ×*g* for 1 h at 4°C. Finally, the pellet was resuspended in 3 ml of the diluent.

#### DiI labeling of exosomes

Exosomes were dissolved in PBS and 0.01mM DiI dye in alcohol was added, and then the solution was incubated for 30 min at 37 °C. The solution was centrifuged at 12,000 ×*g* for 30 min and the supernatant was filtered using 0.2 μm filters. Excess DiI dye was removed by centrifugation at 120,000 ×*g* for 1 hour at 4°C and the pellet (the DiI-labeled exosomes abbreviated as DiI-exo) was resuspended with the diluent.

#### Observation of exosomes

Samples (Part I, the filtered seminal supernatant and DiI-exo) detection and counting were performed on the NanoSight NS300 following manufacturer's protocols (Malvern Instruments, Malvern, UK). Particle concentration (particles/mL) was calculated by the NanoSight system. Samples of exosomes were observed by electron microscopy using previously described methods. The concentration of exosomes in Part I was the concentration of exosomes in the original seminal plasma.

### Semen processing

Sperm pellets were diluted in the diluent (to ∼10^7^ spermatozoa/ml concentration) for the control group (no exosomes, Exo-0), and other groups including Exo-1 (the diluent with 1× exosomes concentration of the original seminal plasma), Exo-4 (the diluent with 4× exosomes concentration of original semen), and Exo-16 (the diluent with 16× exosomes concentration of original semen), respectively. Finally, semen samples (∼10^7^ spermatozoa/ml concentrations) were all stored in the incubator at 17°C±0.1°C in an atmosphere 5% CO_2_ and saturated humidity.

In order to observe exosomes interacting with spermatozoa, the experiments were conducted using the following procedure. Briefly, varying concentrations of DiI-labeled exosomes were added into the diluent. At different time points during sperm incubation at 17°C, the sperm were collected by centrifugation at 800 ×*g*, 10 min at 17°C. Sperm were washed 3 times with the pre-cooled diluent. Finally, sperm in the diluent dropped on a clean slide were observed under fluorescent microscope. At least three separate experiments (consisting of three replicates) were conducted for each concentration of exosomes.

### Analysis of sperm motility

The percentage of motile sperm was assessed visually every 2 days using a phase-contrast microscope (Nikon, Tokyo, Japan), equipped with a warm stage. After 5 min of incubation at 37°C, 5 μl of every semen sample was placed on a glass slide and covered by a glass coverslip. Sperm motility estimations were performed in three different microscopic fields for each semen sample. Sperm motility was evaluated by visual estimation. Ten μL of sperm suspension was delivered onto a pre-warmed clean glass slide, and covered with a clean coverslip. The slides were examined under an optical microscope with a bright field (Nikon 80i; Tokyo, Japan) at 200× total magnification. The percentages of sperm showing progressive movement were estimated and noted after viewing five different fields. Three separate aliquots (replicates) were assessed from each semen sample. The mean of the three successive estimations was recorded as the final sperm motility (%).

### Analysis of sperm membrane integrity

Sperm membrane integrity was evaluated using fluorescent probes [49]. Briefly, The SYBR-14 was prepared in anhydrous dimethyl sulfoxide (DMSO) at a concentration of 0.1 mg/ml. Propidium iodide (PI) was dissolved in PBS at 2 mg/ml. Aliquots (500 μL) of sperm were stained at 37^o^C with 0.27 μL of SYBR-14 and 2 μL of PI solution. The samples were incubated for 15 min at 37^o^C before examination. Sperm staining was monitored and photographed at 400× magnification by an epifluoresence microscope (Nikon 80i; Tokyo, Japan) using filters set to 535 nm excitation and 617 nm emission for PI red fluorescence and 488 nm excitation and 516 nm emission for SYBR-14 green fluorescence. For the same field, photographs were also taken with a phase-contrast microscope. At least 200 spermatozoa per slide were counted. The mean of three successive estimations was recorded as the final sperm membrane integrity score (%).

### Total antioxidant capacity (T-AOC) activity assay

T-AOC activity was determined using a T-AOC assay kit (Nanjing Jiancheng Bioengineering Institute, Jiangsu, China) according to the manufacturer's instructions. The T-AOC activity was measured at 520 nm using a spectrophotometer (Shanghai Spectrophotometer Co., Ltd., Shanghai, China). Finally, T-AOC activity of each semen sample (10^7^ spermatozoa/ml) was converted into units per ml (UI/ml) of total protein in spermatozoa.

### Malondialdehyde (MDA) content assay

Malondialdehyde content was measured with 2-thiobarbituric acid by monitoring the absorbance change at 532 nm with the spectrophotometer (Shanghai Spectrophotometer Co., Ltd., Shanghai, China) according to the instructions of the MDA kit (Nanjing Jiancheng Bioengineering Institute, Jiangsu, China). MDA content of spermatozoa samples (10^7^ spermatozoa/ml) were expressed as nmol/l.

### Sperm capacitation and chlortetracycline (CTC) staining

#### Sperm capacitation

Semen samples were centrifuged (800 ×*g*, 10 min at 17°C) and the pellets were diluted to a concentration of 1.5×10^7^cells per mL in Tyrode's medium (100 mM NaCl, 21.7 mM lactate, 20 mM Hepes, 15 mM NaHCO_3_, 5 mM glucose, 3.1 mM KCl, 2.0 mM CaCl_2_, 1.0 mM pyruvate, 0.4 mM MgSO_4_, 0.3 mM NaH_2_PO_4_, and 50 mg/ml kanamycin, pH 7.4) with 3 mg/L bovine serum albumin (BSA). Samples were then was incubated for 3 h in a humidified incubator in an atmosphere of 5% CO_2_ at 37 °C to induce *in vitro* capacitation [10, 50].

#### Sperm CTC staining

The CTC staining procedure was essentially carried out as described [43]. In brief, CTC (750 pM) was prepared in a buffer of (mM) 20 Tris, 130 NaCl and 5 DL-cysteine (final pH 7.8). Five μL of sperm suspension was mixed with 5 μL CTC on a clean warm slide; after 30 sec 5 μL of 0.2% gluteraldehyde solubilized in 0.5 M Tris buffer (final pH 7.4) was added. One drop of 0.22 M 1, 4-diaza-bicyclo [2, 2, 2] octane dissolved in glycerol:phosphate-buffered saline (9:1) was added to retard the fading of CTC fluorescence. After adding a clean coverslip, 200 spermatozoa per slide were observed using a fluorescence microscope equipped with phase contrast and green epifluorescent optics (Nikon, Japan).

### Western blot analysis

Exosomes pellets were lysed using RIPA buffer containing 1 % protease inhibitor cocktail (Cat NO. BML-KI103-0001, Enzo Life Sciences, USA). Lysates were centrifuged at 14,000 ×*g* for 20 min. Supernatant fractions were electrophoresed on a 10% SDS-PAGE gel and transferred to 0.45-μm PVDF membranes (Millipore, USA). Then the membranes were blocked using 5% (w/v) skim milk for 1 h and washed three times in TBST. The membranes were incubated overnight at 4 °C in a primary antibody solution containing anti-CD9 from goat polyclonal antibody (Cat.No. Sc7639; Santa Cruz), anti-CD63 (Cat. NO. sc-15363, Santa Cruz) and anti-AWN (see Table S1 and Figure S1) at a dilution of 1:1000. After washing with TBST, the membrane was incubated in the goat anti-rabbit IgG (or the monkey anti-goat IgG for CD9) coupled to horseradish peroxidase (1:10,000) and for 1 h at room temperature. The membrane was then incubated 5 min with ECL and chemiluminescence was detected using the BIO-RAD ChemiDoc XRS Imaging system.

### Indirect immunofluorescence

Sperm samples were washed three times with PBS at 800 ×*g*for 5 min at room temperature. Sperm suspension (1×10^7^sperm/ml) was fixed in 4% (w/v) paraformaldehyde (PBS/PFA) for 30 min. After washing three times with PBS, the sperm were resuspended in PBS and placed onto poly--lysine-coated coverslips (5-μL sperm suspension/spot), and completely dried for 15 min at room temperature. The sperm spots were then washed three times with PBS before immunofluorescence staining. Briefly, sperm spots were incubated for 30 min at 37°C in PBS supplemented with 2% skim milk (vlv). Spermatozoa were incubated overnight at 4°C with the primary antibody to AWN and PSP-1 (see Table S1 and Figure S1). Identical samples incubated in parallel in PBS supplemented with rabbit preimmune serum were used as negative controls. After overnight incubation in primary antibody, the slides were washed extensively with PBS and incubated for 30min at 37°C with the secondary antibody (FITC-conjugated polyclonal antibody to rabbit IgG). The slides were then incubated with DAPI for 2 min. Finally, the slides were washed three times with PBS, and examined using a fluorescent microscope.

### Scanning electron microscopy

Two mL of sperm sample were fixed in 2.5% glutaraldehyde in 0.01 M phosphate buffered saline buffer (PBS) for 1 h at 4°C. After the fixation, the samples were centrifuged at 800 ×*g* for 2 min and then washed three times with PBS. Following mounting on silicon slice and air drying, sperm samples were post-fixed in 0.2% aqueous osmium tetroxide for 30 min and then dehydrated in increasing concentrations of acetone (35, 50, 75, 95 and 100%) was followed by critical point drying. One mL of exosome solution was directly dropped onto a silicon slice and air dried. Finally, the specimens were sputter-coated with gold palladium and viewed under a scanning electron microscope (Leo 1455 VP, Cambridge, UK).

### Statistical analyses

All analyses were performed using Statistical Product and Service Solutions (SPSS 11.5 for windows; SPSS, Chicago, IL, USA). All results were expressed as mean SD. The mean values of the percentages of motile sperm, dead sperm, plasma membrane-intact sperm and T-AOC level and MDA content were compared using Duncan's multiple range tests by ANOVA procedure, when the F value was significant (*p* < 0.05). The correlation among the above-mentioned methods was evaluated by linear regression analysis.

## SUPPLEMENTARY MATERIAL FIGURE AND TABLE


