# Supplementary material for: Boar seminal plasma exosomes maintain sperm function by infiltrating into the sperm membrane
Source: Oncotarget. 2016 Aug 16;7(37):58832–47. doi: 10.18632/oncotarget.11315 (PMC5312279; doi:10.18632/oncotarget.11315)
Supplement: Supplementary file 1 [file oncotarget-07-58832-s001.pdf]

Boar seminal plasma exosomes maintain sperm function by infiltrating into the sperm membrane

Supplementary Material

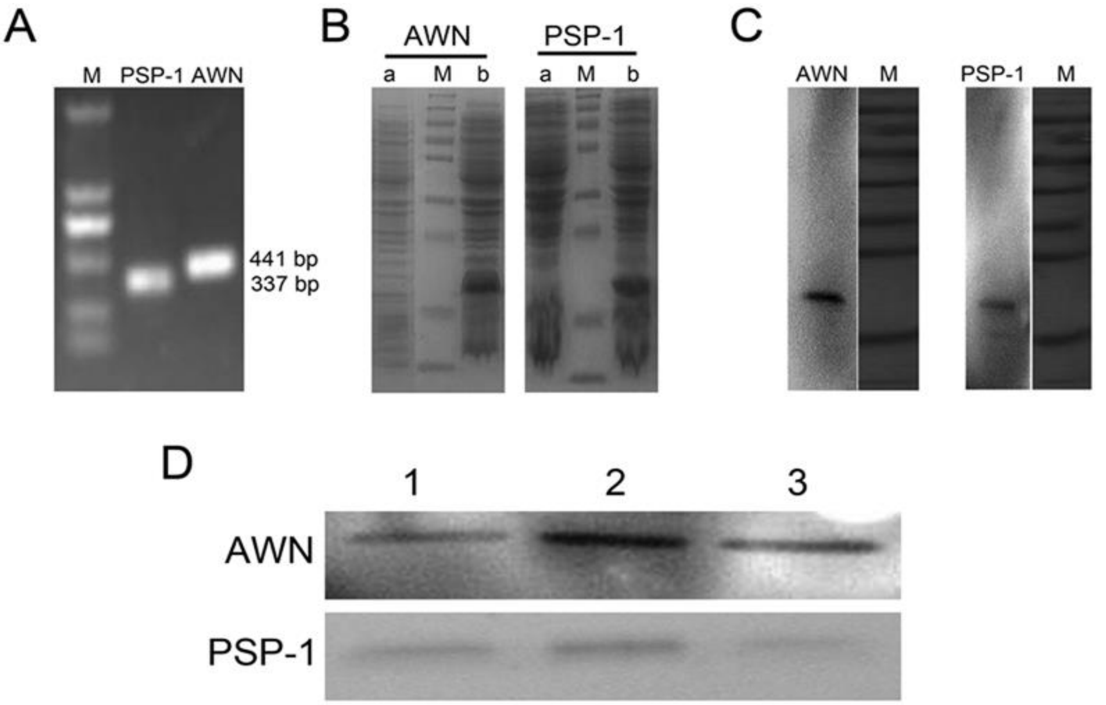

Supplementary Figure S1 Preparation of anti-boar AWN and PSP-1 rabbit derived polyclonal antibody

Boar AWN and PSP-1 anti-rabbit polyclonal antibody were prepared on the basis of a normal method as report of Chun-Fang Zhu et al [1]. Briefly, boar AWN (NM_213829) and PSP-1 (AF427041) genes were amplified from boar testis cDNA using primer sets (see Supplementary table S1). Boar AWN and PSP-1 were directly cloned into plasmid PET28a and obtained the recombinant plasmids (PET28a-AWN and PET28a-PSP-1). After expression of recombinant proteins were induced by IPTG in *E.coli.*, the supernatants containing recombinant proteins were subsequently purified by affinity chromatography on nickel–nitrilotriacetic acid Superflow resin (Qiagen, Germany). Purified recombinant proteins were emulsified with equal volumes of Freund's complete adjuvant (Sigma) for the first injection into rabbits and Freund's incomplete

adjuvant (Sigma) for the next three booster immunizations. The Rabbits received the four subcutaneous injections at one-week intervals. Anti-sera were collected at the 7th day after the last injection. The anti-sera were tested by ELISA for specificity to the expressed AWN and PSP-1 proteins, respectively.

Preparation of AWN and PSP-1 antisera was shown in supplementary Fig. S1. A，AWN and PSP-1 CDs. B, Expression level of AWN and PSP-1 protein before (b) and after (a) IPTG inducing. C, Purification of AWN protein and PSP-1 protein by nickel–nitrilotriacetic acid Superflow resin. D, AWN and PSP-1 proteins in boar seminal plasma were detected using these rabbit antisera (contain with AWN and PSP-1 polyclonal antibody) by western blot, respectively. 1, 2, 3 represented three boar seminal plasma samples.

Supplementary Table S1 Primers in this study

Gene	primers	annealing temperature (^{o}C)	Gene ID
AWN	ATGGGTCGCGGATCC<u>**GAATTC**</u>GCATGGAACAGAAGGTCTCGTTCCTGTGGCG<br>CTCGAGTGCGGCCGC<u>**AAGCTT**</u>AGGGATGTTTTTCTCTGTAG	59	GI:47523193
PSP-1	ATGGGTCGCGGATCC<u>**GAATTC**</u>TGGGGCTTGGACTATCATGCCTGTGGTG<br>CTCGAGTGCGGCCGC<u>**AAGCTT**</u>ACCTTGTGAGTCACGAAG	59	GI:47523175

Reference

1. Zhu CF, Liu Q, Zhang L, Yuan HX, Zhen W, Zhang JS, Chen ZJ, Hall SH, French FS, Zhang YL. RNase9, an androgen-dependent member of the RNase A family, is specifically expressed in the rat epididymis. Biol Reprod. 2007; 76:63-73.
